# Reasoning “Uncharted Territory”: Notions of Expertise Within Ethics Review Panels Assessing Research Use of Social Media

**DOI:** 10.1177/1556264619837088

**Published:** 2019-12-12

**Authors:** Chelsea Sellers, Gabrielle Samuel, Gemma Derrick

**Affiliations:** 1 University of York, UK; 2 King’s College London, UK; 3 Lancaster University, UK

**Keywords:** social media, research ethics committee, ethics, experience, expertise

## Abstract

The fast changing field of social media (SM) research presents unique challenges for research ethics committees (RECs). This article examines notions of experience and expertise in the context of REC members reviewing proposals for SM research and considers the role of the RECs in this area of review. We analyze 19 interviews with REC members to highlight that a lack of personal and professional experience of SM, compounded by a lack of institutional and professional guidelines, mean many REC members feel they do not possess sufficient expertise to review SM research. This view was supported by 14 interviews with SM researchers. REC members drew on strategies to overcome their lack of experience, although most SM researchers still found this problematic, to varying degrees. We recommend several steps to ensure REC expertise in SM research keeps pace of this fast-developing field, taking a pro-active, dialogic approach.

## Introduction

There has been an increase in recent years in the use of social media (SM) for research purposes, in part due the increase in the range and use of such platforms and the variety of data they produce (Bassett & O’Riordan, 2002; Carter et al., 2016; Zeng, Chen, Lusch, & Li, 2010), resulting in a research agenda that has “drawn attention from research communities in all major disciplines” (Zeng et al., 2010, p. 14). This increased use of SM data for research purposes raises a range of new ethical challenges. Debate has centered around several key issues relating to whether SM data should be considered human subjects research or published data, whether SM users have perceived expectations of privacy when using SM platforms and how this influences practices of consent (is consent required, is it feasible?), and issues of confidentiality and identifiability of participants (Bassett & O’Riordan, 2002; Carter et al., 2016; Henderson, Johnson, & Auld, 2013). Given these challenges, many scholars feel that SM research complexities traditional research ethics frameworks, such as those proposed by Emanuel, Wendler, and Grady (2008), who outline eight ethical principles which commonly underpin institutional and professional ethical guidelines. These principles include collaborative partnership, social value, scientific validity, fair selection of study population, favorable risk–benefit ratio, independent review, informed consent, and respect for recruited participants and communities (Emanuel et al., 2008; Emanuel, Wendler, Killen, & Grady, 2004). Henderson et al (2013) argue that, although traditional principles “continue to be relevant and valuable, [. . .] there needs to be a more nuanced understanding of how they apply in new and emerging technology-mediated social spaces” (p. 547).

Scholars and professional bodies have published guidelines to aid SM researchers and research ethics committees (REC; in the U.S. Institutional Review Boards [IRBs]) as they try to navigate this new and complex ethical terrain (The British Psychological Society, 2013; British Sociological Association, 2017; Markham & Buchanan, 2012). The guidelines—noting the fast-developing nature of SM, and the variety of unique SM platforms available to research—purport that developing specific, blanket ethical principles for SM research is difficult and undesirable. As the British Sociological Association (BSA) highlight in their recently published statement of ethical practice for digital research:

We should not necessarily rule out digital research that does not conform to ethics processes originally designed in a very different context, nor can we provide guidelines that encompass all forms of digital research that may become possible in future. Each research situation is unique and it will not be possible simply to apply a standard template in order to guarantee ethical practice. (BSA, 2017, p. 11)

These guidelines advocate a researcher-led, case-based approach to ethical decision making, rather than strict guidelines on how to conduct such research ethically (Samuel, Derrick, & Van Leeuwen, 2019). Although this seems the most appropriate approach with which to consider ethical decision making for SM research, guidelines have been described by researchers as both limited and insufficient (Samuel et al., 2019; Woodfield et al., 2013).

When reviewing SM research, RECs need to negotiate this lack of guidance, or consensus regarding the ethical implications of SM research, and the fact that traditional ethical principles (Emanuel et al., 2008) may no longer apply or may need adjusting. Although there have been calls to provide more REC training in this area to help REC members negotiate these challenges (Buchanan & Hvizdak, 2009; Woodfield et al., 2013), SM researchers rarely publish how they negotiate the ethical challenges they face (Ienca et al., 2018; Samuel et al., 2019) and are not always required to submit research for ethical review, offering little in the way of passing on knowledge to RECs in this area (Henderson et al., 2013).^1^ Previous research suggests that when there is a lack of clear guidance or knowledge during REC group ethical decision making, REC members use justifications (or “warrants”) based on their professional or personal experiences, which must then be accepted by the group as sufficient to support their judgment. When REC members lack warrants, a process of deliberation, which welcomes researcher input, is required (Stark, 2011).

Given the ethical challenges of SM research, and given the lack of clear ethical guidance in this area, using an interview methodology, we wanted to explore which warrants REC members draw upon when reviewing SM research proposals. We were interested in the influence of these warrants on their decision making, and on the decision making of SM researchers, and the implications of this more broadly in terms of REC review of SM research proposals. Previous scholarship has explored REC members’ abilities to act as experts when reviewing research in which they lack direct experience, as well as the implications of this in terms of the value of RECs as a mode of ethics governance more broadly (Van den Hoonaard & Hamilton, 2016). Given this, we turned our attention away from our exploration of the nature of “warrants” to explore these issues further, considering the role of RECs in reviewing SM research proposals.

In the following section, we provide a brief review of the literature exploring the role and nature of expertise in RECs. We then document our interview methodology before presenting our findings. The findings explore REC and researcher perceptions of REC experience and expertise in relation to SM research and the implications of this for the ethics review for SM research.

## RECs and Expertise

RECs are a central feature of ethics governance in many Higher Education Institutions, but their necessity and value, particularly in the social sciences, is still questioned (Birnbacher, 2012; Douglas, 2012; Emmerich, 2015; Rasmussen, 2016; Van den Hoonaard & Hamilton, 2016). One of the many prominent concerns relates to RECs’ lack of experience and/or expertise when reviewing research in certain (social) scientific fields which use specific methodologies, or have “untraditional” ethical concerns which need addressing (Birnbacher, 2012; Douglas, 2012; Emmerich, 2015; Iltis & Sheehan, 2016; Rasmussen, 2016). Others, such as Garrard and Dawson (2005), have challenged these critiques, arguing that the legitimacy of RECs derives from the diversity of their members and the process of deliberation they engage in and, therefore, “there are no more grounds to worry about the legitimacy and authority of RECs than of any other similarly constituted regulatory body” (p. 423) or similarly fallible human institution.^2^ As is also noted below, RECs have strategies in place for situations where members’ collective lack of experience may call this legitimacy into question.

However, perceived issues remain regarding REC experience/expertise because the terms “experience” and “expertise” are often used interchangeably, although experience is arguably an overly simplistic measure of expertise (Hapeshi, 2014). To improve ethics governance within Higher Education Institutions—or perceptions of this governance, more thought is needed regarding how these terms apply in the context of REC decision making. Hapeshi (2014) argues that expertise is better viewed as being socially constructed, that is, as dependent on how others view a REC member and whether or not they value them as an expert. Collins and Evans offer an alternative conceptualization (Collins & Evans, 2007). For these authors, to be considered an expert capable of making judgments and engaging in dialogue within a particular field, REC members must have at the minimum “interactional” expertise, that is, not necessarily being a researcher within a field, but being able to discuss a research field due to familiarity with the relevant literature. At a maximum, REC members must have contributory expertise—the “traditional way of thinking about expertise” (p. 24), acquired through firsthand experience in a field. This necessity for expertise is crucial if a REC member is to make ethical judgments on a research proposal in a particular scientific field (Savulescu, 2017). Sirotin and colleagues found that REC Chairs reviewing mental-health-related research draw on colleagues with appropriate scientific expertise for help when reviewing proposals for which they have little experience (Sirotin et al., 2010). Hammersley notes, “sound ethical judgment, like good practical research decision-making, demands contextual knowledge” (Hammersley, 2009, p. 220); and Ienca and colleagues stress the need for awareness of the methodological challenges presented by new forms of health data research, highlighting the interconnection between ethical, methodological, and technical issues (Ienca et al., 2018, p. 9). Therefore, although it is important to note that RECs must in the first instance draw on research ethics principles as opposed to their understanding of the research topic/ method in question, REC members must be mindful of the ethical implications of new forms of research and the ways in which they might problematize traditional frameworks.

REC members cannot, however, have contributory, or even interactional, scientific expertise in all fields of research which require ethics review (Dove & Garattini, 2018). Indeed, most RECs frequently have to review novel research approaches or research using novel technologies—this is not an issue which is unique to SM research (Milford, Wassenaar, & Slack, 2006; Mutenherwa, Wassenaar, & de Oliveira, 2018). For example, Mhaskar conducted a nationwide survey of IRB members from major U.S. research universities focusing on basic knowledge about clinical research study designs and found significant knowledge deficits within committees (Mhaskar et al., 2015). Dove similarly identified a lack of REC member expertise in data-intensive science, including the challenges it presents to traditional notions of “specific” consent and the need to link data to multiple datasets (Dove & Garattini, 2018), and Buchanan and Ess’s (2009) extensive survey of U.S. IRBs has previously suggested that members often feel underprepared to evaluate study protocols involving SM research methods (Buchanan & Ess, 2009). Most recently, Ienca and colleagues highlight the challenges posed for RECs by the new and complex nature of health-related big data (Ienca at al., 2018, p.1). In contrast, a recent U.K. survey found little issue with REC member experience of SM research, but was conducted at primarily one institution and cannot be considered as representative (Carter et al., 2016).

For some, REC members’ lack of contributory expertise is unproblematic since an important purpose of committees is to foster a diversity and range of expertise so that members who lack expertise in one field can defer ethical judgments to other committee members (Dove & Garattini, 2018). As Hapeshi’s findings suggest “committee members see themselves as part of a team, with individual members making different contributions to a collective task. Viewing REC members in this way allows their different expertise to be formally recognized” (Hapeshi, 2014, p.v). It is also important to remember that, other than in the case of an expedited review or where chair’s action is taken, decisions rarely rest on the experience of a single member, rather recommendations are made by individual members and decisions are made by the REC as a whole. Moreover, it could be argued that in situations in which no committee member has the relevant contributory expertise, REC members will already be well-versed interactional experts in how to apply “standard, traditional” ethical principles for these fields of research, which should be considered in the first instance.

Others find this scenario more problematic since some research fields, which complicate traditional standards of ethical practice (Emanuel et al., 2008), raise issues for which REC members may be ill-equipped to negotiate, and their interactional expertise may no longer apply. Savulescu has called for more contributory expertise via superregional specialist ethics committees “with people with the right skills and experience to identify risks and to engage in ethical reflection and deliberation” (Savulescu, 2017, p. 2). Goodyear-Smith and colleagues propose, improving interactional expertise in other research areas. In relation to implementation science, they call for educating committee members on the nature of such research, including “researchers explicitly mentioning these issues in their application; supplying a key paper outlining this type of research as supplementary material; or seeking the opportunity to meet with the committee and explain co-design in the context of their proposal” (Goodyear-Smith, Jackson, & Greenhalgh, 2015).

SM research is a research field which complicates traditional standards of ethical practice, such as those outlined by Emanuel at al. (2008). For example, McKee (2013) highlights the ways in which SM such as Facebook and Twitter blur the boundaries between public and private, with traceable, publicly available data problematizing notions of anonymity, informed consent, and what might constitute “human” research—issues which are compounded by what is often an absence of direct communication between researchers and participants. Issues surrounding privacy and consent, in addition the implications of user expectations of SM with regard to usage of their data, have also been highlighted as ethical challenges in SM research (Conway & Connor, 2016). Hibbin, Samuel, and Derrick (2018) also found that the public availability of SM data problematized traditional ethical principles, advocating for a more “nuanced approach to data use and consent” (p.149) within RECs. Little research has explored notions of experience and expertise in this context, how they play out when RECs review SM research and what this tells us about the role of the REC in reviewing SM research. The following research questions are addressed in this article to explore these issues:

1. What is the association between REC experience and their level of expertise in relation to the ethics of SM research, as perceived by
  - REC members
  - SM researchers
2. What impact does this have on the decision making of RECs regarding the ethical implications of SM research?

This article reports REC member experience and expertise reviewing SM research, and discusses the implications of these findings in terms of the conceptualizations of expertise highlighted above, as well as in terms of ethical REC governance of the SM research field in general.

## Method

### Recruitment and Sampling

#### RECs

Sixty-three REC Chairs and/or members at the 20 most research-intensive U.K. universities^3^ (as determined by the U.K. Research Excellence Framework 2014) were identified via university websites and contacted, 19 of whom responded and participated (nine Chairs, one deputy Chair, nine members). This means that our response rate was 29%, which, although low, is within the normal parameters for qualitative research.

Participants represented 13 U.K. institutions across 18 different university-level or faculty/department-level RECs. Ten participants sat on a university-level REC, and 13 participants sat on a departmental/faculty-level REC (four participants sat on both). REC members came from a range of disciplinary backgrounds (education, psychology, sociology, philosophy, psychiatry, history, health, and politics), however, no differences in interviewees’ experiences of SM research and their perceived expertise were highlighted in their narratives.

#### Researchers

An in-depth bibliometric search for publications using U.K. SM research data was conducted to identify U.K. researchers using SM data (*N*=147). After data-cleaning, from these, 14 researchers participated in the interviews. Scholars were from psychology, computer science, informational systems, Science, Technology, and Society (STS), anthropology, linguistics, and public health, and were experienced with using a broad range of different qualitative, quantitative, and modeling methods.

### Interviews

Interviews were semi-structured. G.S. conducted recorded interviews (40-60 min) either face-to-face, over the telephone or via Skype. Interview schedules were constructed to answer the research questions. The interview schedule for REC interviews asked participants about their own experience of using SM data for research, as well as their use of SM more generally in their professional or personal life. The questions also explored interviewees’ views about the ethical issues surrounding the use of SM data in research, their knowledge about the policies at their own institution in relation to this, their experiences of reviewing such research in a REC capacity, and their decision making in relation to this. We also asked about any guidelines, training, or literature they had used to aid their decision making in this area and, for those with no experience in this area of ethics review, how interviewees thought they would make decisions about this research in their capacity as a REC Chair/member.

The interview schedule for researchers similarly explored interviewee’s views and experiences about the ethical issues surrounding the use of SM data for research and knowledge about the policies at their own institution in relation to this. We also explored researcher’s views about whether such research should require ethics approval, whether they choose to have their research reviewed, and any guidelines they referred to, to aid their decision making in this area.

Interview schedules can be made available upon request.

### Analysis

Data were analyzed using a grounded theory approach and inductive reasoning (Charmaz, 2006; Strauss, 1987). The analysis and coding of the data was completed in two stages: overview analysis and detailed analysis. Overview analysis consisted of memo-making. This phase was conducted in duplicate by the authors. Lengthy discussion post memo-making pointed to relevant themes of interest, and a detailed analysis of the data, the results of which are detailed in the following section.

## Results

In the following section, the results of our analysis are outlined and discussed. We begin by addressing the themes arising from the REC interviews. We consider the implications of REC members’ limited personal and professional exposure to SM on the extent to which they felt warranted to make judgments on the ethics of SM research. We then highlight how this is compounded by a lack of clear formal guidelines from both institutions and professional bodies, and outline the strategies REC members use in lieu of such guidelines.

We close this section by turning to the perspectives and experiences of the SM researchers interviewed—highlighting the implications of REC members’ lack of experience for those going through the review process, and its impact on the perceived purpose of RECs and the ethics review process. The broader implications of these findings, in terms of our research questions and practice in this area, are then considered in the conclusion to the article.

### Lack of Professional and Personal Warrants

REC members had limited exposure to reviewing SM research proposals and some had never previously encountered such a proposal (*n* = 6/19). As such, many interviewees had not thought deeply about the ethical issues related to such research. Some interviewees had tried to address this, attending workshops and training events. However, although viewed as useful, there was an implicit suggestion by some REC members that such training did not always equate to being able to make their own decisions in their role as REC members. For example, for REC Member 1, despite being a member of the Association of Internet Researchers and having some awareness of the ethical issues surrounding SM, they were “mostly an observer” in such discussions and did not claim to possess any degree of relevant expertise.

In cases where REC members have no professional experience, Stark and others state that members will draw on personal experience during ethical decision making (Fitzgerald, Phillips, & Yule, 2006; Stark, 2011), but interviewees’ lack of exposure to reviewing SM research proposals was compounded by a lack of both research and personal experience of using SM platforms. In fact, only a minority of interviewees (*n* = 5/19) explicitly acknowledged interacting with SM platforms for personal reasons and only five participants reportedly used SM data for their own research. Many interviewees were therefore also unable to draw upon such experience of SM platforms to aid with their decision making. Five of the REC members spoke about a generational gap between research students/early career researchers and more senior academics, who were perceived to be less likely to use SM in either a personal or professional capacity. REC Member 3 described himself as “too old” to use SM and questioned whether they were the “right person” to comment on the use of SM data for research. In contrast, younger scholars were perceived to be more likely to use SM in research (“it’s mostly students using social media to collect data” REC Member 18) and dissemination of research findings:

I mean, you look at public engagement side of things, and that’s where you see the link the use of SM technology. That’s mainly done by research associates or younger academics, less so by the PI. So I think there’s something there which is perhaps a generational thing. (REC Member 8)

Some REC members suggested that this limited personal and professional exposure to SM positioned them as unable to provide valuable insights into the topic of the interview—exploring REC members’ perceptions of the ethical issues related to SM research. For example, in response to a question regarding their experience of SM in their role as a researcher, REC Member 1 considered whether they were a suitable participant for this study:

I might be in fact the worst person to interview but—because I don’t have any social media account. I never use social media account. [. . .] Obviously, I know Facebook and Twitter—and what’s that the one that you share pictures—Instagram. But kind of like those things are the ones that I only know from day-to-day life. I never use any of them; I never did any research on them. (REC Member 1)

REC Member 2 made similar comments regarding their suitability as an interviewee, stating that “I’m the wrong person for you to—to be enrolled in this, really. I have absolutely nothing to do with social media.”

These findings raise questions about which warrants our interviewees drew upon during their decision making in the absence of professional and personal warrants. REC members’ reluctance to assert expertise in this area, and the tentativeness of their answers illustrates a degree of uncertainty regarding the ethical issues arising from such research. As several interviewees suggested, their lack of familiarity with the intricacies of such platforms and the conditions in which the data have been created meant that they lacked the requisite personal and professional warrants to make informed judgments on the ethics of such research. Although there is an increasing number of ethics applications from SM researchers, the overall number of SM research proposals is still relatively low, so REC members remain inexperienced. This is problematic considering the rising number of applications coming through for review, as RECs do not yet have the prior experiences on which to base their judgments.

### Lack of Ethical Guidelines for SM Research

Interviewees reported a lack of useful ethics guidelines specific to SM research to aid them with their ethical decision making. This left no set precedent (or professional warrant) to fall back on for those who lacked experience in this area. This, compounded by the fact that researchers rarely report on the ethical issues arising from such research (Henderson et al., 2013; Ienca et al., 2018; Samuel et al., 2019)^4^ and the fact that SM is a new, complex and fast changing phenomenon, made it difficult for REC members to know how to make appropriate decisions about SM research.

In most cases, institutional guidelines were either non-existent or in the process of being developed and implemented, often comprising “occasional paragraphs in our existing guidelines” (REC Member 11). Although interviewees did note that the development of guidelines was an “ongoing process” (REC Member 11), prompted by a rise in the number of such applications. These findings are in line with those from the ethics guideline analysis strand of this project, which highlighted that 18 out of 20 universities did not have ethics guidelines for SM research (although four were in the process of developing guidelines, and six were aware that they were needed, or directed researchers to external guidelines) (Samuel, Derrick, & Van Leeuwen, 2019).

Beyond institutional guidelines, interviewees spoke about SM guidelines published by various professional bodies—for example, the Association of Internet Researchers (Markham & Buchanan, 2012) and The British Psychological Society (2013). Some interviewees placed heavy weight on these documents to make up for their lack of personal and professional experience of SM. For example, REC member 16 felt that this guidance (and the low number of SM proposals they are faced with) meant they were confident in reviewing SM research proposals despite their lack of personal and professional experience:

I do feel overall, yes, that we were in a position to be able to respond. I don’t think for every situation potentially but given the low numbers that we get I’m not sure that we necessarily be caught out or faced with a challenging or situation that guidance couldn’t help us work through. (REC Member 16)

However, most interviewees, echoing the literature (Hibbin et al., 2018), described the guidelines as “vague.” In contrast to less dynamic, more homogeneous fields, this vagueness was perceived by nearly half of the interviewees (*n* = 8/19) as something which could not be rectified for SM research. Rather than referring to specific platforms and the different challenges they present, it was this constant stream of new, constantly evolving SM platforms which made keeping up with the ethical implications of SM research difficult. There was an awareness that the guidelines needed to remain flexible enough to be applicable to a variety of SM platforms, both now and in the future. REC Member 1, for example, perceived little purpose in the process of developing guidelines which will expire no sooner than RECs have caught up with the latest developments. Consequently, some REC members question whether it is even desirable to enforce a set of restrictive guidelines onto all SM research if these guidelines will change as technology advances;

if you enforce something, then it’s going to be completely irrelevant in some other context and then it’s going to cause a trouble rather than help you. But it may not be helpful in two years, so you just have to sit down and think about all the ethics. (REC Member 1)

Although in some cases formal guidelines were considered useful for inexperienced REC members, most interviewees recognized that for the field of SM research, guidelines could only partially compensate for REC’s lack of experience and perceived expertise with regard to SM research.

### Strategies to Aid Decision Making

When unsure about the ethical implications of SM proposals, some interviewees spoke about educating themselves in this area; “when I first started, I hadn’t much experience in that, so it’s also trying to research what the issues are” (Interviewee 17). A number of them also spoke about SM ethics training days which had been implemented in some of their institutions: “we invited her [an expert in SM] to speak to our research committee . . . it was our annual training day” (Interviewee 5).

Interviewees also deferred ethical judgment to those REC members more experienced than themselves. Interviewee 10 suggested that when faced with the task of reviewing a proposal for research which uses platforms they are unfamiliar with, they would not feel confident responding without seeking advice from someone else. This is because they would not be aware of the potential ethical consequences of using the platform for research purposes due to the newness and fast changing nature of the platforms. Although they did not see this as an issue, as they note that an awareness of this gap in knowledge, and the fact that it is a “known unknown,” means that they can take steps to address this:

So, I think that’s certainly a big concern and I just don’t know if I got an application from someone who wants to use dark web I would have to take advice, I wouldn’t know quite what I was judging, but you know, I know I don’t know so it’s a kind of a known unknown.

This decision to seek advice was echoed by many of the other interviewees, who spoke about the importance of knowing when to seek out expertise. Such expertise could be gathered from other members of the REC committee, or from RECs in other departments or faculties:

My understanding of all our committee members is that they have enough insight to know that obviously is this something that they are dealing with that they are not aware of. They can ask for another member to take over or ask for guidance, the next we’ve got people from the data management and big data groups and things like that. (REC Member 17)

Interviewees explained that working on a case-by-case basis and dealing with issues “after the fact” is generally used by REC members when faced with any issue or methodology which they are unfamiliar with. The hope was that this approach, incrementally drawing on lessons learned from previous experiences when considering present applications, would lead to shared understandings and more formalized norms of ethical practice, building interactional expertise. As Interviewee 11 suggested, once REC members have increased experience of reviewing SM research and they have built a knowledge base regarding potential ethical issues of SM research, ideas regarding what constitutes best practice will be solidified:

I think more likely, step-by-step that some of the things that we decided on case-by-case basis may become more formalized, such that we can actually can give people advice of this would be acceptable, this wouldn’t or have you thought about something else. And I think that just comes from the committees having more experience.

Thus, in line with previous literature (Sirotin et al., 2010), our findings suggest RECs have strategies in place to develop their interactional expertise—by identifying experts in the field and working on a case-by-case basis.

## Researcher Views

Findings from SM researchers highlight the implications of the findings above for those who are under review. This is important as, in line with the social construction of expertise discussed earlier, an individual (REC member) can be defined as an expert only if they are perceived by others to be so. Only two researcher interviewees felt REC members had sufficient experience with reviewing SM research proposals. One of whom, Researcher 3, was also a REC member and believed RECs are now more familiar with, and have a better understanding of the ethical implications of using SM data for research:

Certainly, with ethics committees, when I started in 2004, there was [ . . . ] this assumption that you can’t possibly do that, it’s just wrong. When asked why, there was no explanation. But nowadays, it’s become more and more—it’s become more and more understandable and maybe people are familiar with it [the use of SM data for research].

For others, who perceived there to be a lack of experience within RECs, the extent to which they perceived REC member inexperience with SM research as problematic varied. For several interviewees, it was unproblematic. For them, REC members were not required to possess interactional expertise since researchers were equally responsible for thinking through the ethical issues related to SM research. Here, in a process of collaboration and negotiation with the researcher, RECs could use their ethical expertise to tease out and predict any ethical concerns which needed to be addressed. The review process for them was therefore an opportunity to engage in a dialogue with the REC, and a mutual exchange of knowledge (Holland, 2016). Researcher 9 describes ethics review as something they do with the REC, rather than something that is done to them. This is a useful process in which the researcher receives and responds to feedback, requiring them to thoroughly think through the issues and deepen their own understanding: “I mean stepping back, the way that we would negotiate any ethical issue is in collaboration with the ethics board. So putting it together, submitting it . . . ”

Researcher 7 also explained that it is not feasible to expect REC members to be experts in every kind of research, but that the process is about communicating and answering questions to work through issues and address concerns the REC member might have:

I think you can’t guarantee that any research ethics committee will have expertise in the kind of research you do, I think it’s kind of naïve to expect that but in my experience, research ethics committees will ask researchers to explain—you know, they might have to ask the question. And I think it’s okay to ask questions as a research ethics committee and you know, and then researchers can explain why they think this approach is appropriate rather than that approach, you know, in the face of any kind of issues that the ethics committee might be concerned about.

In contrast, the majority of the researchers interviewed expressed reservations about RECs’ perceived lack of experience with reviewing research using SM data.^5^ For example, although Researcher 2 understood that researchers should be required to think through the ethical issues relating to their research on their own and then engage in a degree of open dialogue with the REC, this exercise was seen to only be worthwhile when members have sufficient experience for their judgments to be warranted. Without sufficient experience, REC member comments were viewed to be irrelevant and, ultimately, unhelpful:

The committee that’s looking at it [the proposal] should include people with expertise in social media research or at least knowledge of the issue because otherwise you can end up getting comments and requirements and ethical requirements that are meaningless or unnecessary, which has certainly been the experience of well, myself and certainly my colleagues, even more than me.

This perceived lack of experience and familiarity was particularly problematic for Researcher 6, who felt “quite a novice researcher in a way” and “just wanted someone else to have looked at the[ir] research to confirm ‘that’s an ethical approach’”:

I’m not sure it’s different with social media research because the ethics boards aren’t experts in it because it is so new so you kind of feel why do I need to justify it to them when they don’t know what’s going on here. (Researcher 6)

RECs’ perceived lack of experience reflected a lack of ability to be able to reassure this less experienced researcher that they were carrying out their research in an ethical way, as well as to provide what they viewed as a useful or credible process. Their perception of the REC was not one of “experts” whose judgment they could trust. Researcher 10 provides an example of how this lack of inexperience manifests in terms of the ethics approval process as they describe their negative experiences with their REC (see Note 3). Here, they describe conflicting views over whether SM research should be viewed as human participant research and whether such research requires ethics approval:

it just comes back to them saying they can’t review this because it doesn’t contain any people or there’s some very sort of negative response to the request for a review, and that’s why it had all been held out. And so that was my kind of first experience in terms of ethics, that the department and their review system really didn’t know how to handle those kinds of ethical applications. (Researcher 10)

Overall, most researchers viewed REC members as inexperienced—although they varied in the extent to which they viewed this as problematic. At best, this perceived lack of experience was unproblematic as the review process was viewed as a collaborative process between the expert in SM (researcher) and the expert in ethics (REC member); at worst, researchers thought this equated to a lack of expertise and impacted negatively on their confidence in the ethics review process.

## Conclusion

The first research question we sought to address was related to the implications of REC experience of SM for the extent to which RECs were viewed to possess expertise on the ethics of SM research by REC members themselves and SM researchers going through the review process. REC members are responsible as experts in research ethics (Moore & Donnelly, 2015; Stark, 2011). In line with Collins and Evans (2007) theory of expertise, REC members, at the very least, need to have “interactional expertise” to be able to engage in meaningful dialogue with each other and with researchers and to make expert judgments. This interactional expertise rests on a degree of indirect experience—experience reviewing similar applications, familiarity with the literature, training in the ethics of SM, or discussions with researchers. Our findings demonstrate that currently many of our REC members lacked interactional expertise and therefore could not be considered “experts” in SM research ethics. However, at least some REC interviewees did speak about strategies in place to increase their interactional expertise via interacting with SM research experts when required.

Alongside Collins and Evans’ understanding of the relationship between experience and expertise, we can also consider “expertise” as socially constructed in terms of how groups identify experts (Hapeshi, 2014). In this construction of expertise, a REC member “becomes” an expert through the eyes of researchers. Here many researcher interviewees perceived REC members as lacking experience and, in turn, did not consider them experts in SM research. This is problematic insofar as it could ultimately lead to a lack of researcher trust in REC expertise to conduct their role in reviewing SM research proposals, and possibly more broadly. Indeed, a wider critical narrative already exists of the waning lack of trust researchers have for RECs (Guillemin & Gillam, 2013; Van den Hoonaard & Hamilton, 2016). The implications of this are important since RECs need to be viewed as experts in order for the review process to be considered meaningful and relevant (Hammersley, 2009). This is despite the fact that the role of the REC is to engage in a dialogue with researchers (Economic and Social Research Council, 2015; Holland, 2016; Stark, 2011), meaning that the responsibility to be aware of the ethical implications of research is not just in the hands of the committee. Furthermore, the lack of REC expertise in SM research is understandable, and arguably unavoidable considering the pace with which the field is moving.

Our second research question considered the implications of this for the ethics review of SM research and the role of the REC in this process—how did RECs and researchers negotiate this perceived lack of expertise during the review process? As discussed above, at least some REC interviewees spoke about seeking out SM researchers when required, but this approach needs to become more extensive. This is with a view to answering previous calls to understand the relationship between RECs and researchers as collaborative, and to realize that most of REC expertise originates from researchers themselves, through discussing and justifying their own research with RECs (Holland, 2016; Stark, 2011). In this model, REC members must acknowledge that “what it means to be ethical is defined in researcher’s communities of practice” (Holland, 2016, p. 366). RECs must therefore realize the importance of engaging and listening to researchers. Similarly, researchers must be open to understanding that RECs cannot realistically be expected to understand all of the issues related to SM research, and that REC expertise will be gained only from dialogue with researchers working in the field. As Collins and Evans (2007) note, interactional expertise feeds on the contributory expertise of others. Indeed, our findings suggest that our interviewees are already using such a strategy. Elsewhere, we have also argued for all SM research proposals to be submitted for REC review so REC members can quickly gain the interactional expertise necessary to be able to make ethical judgments on this research (Samuel et al., 2019).

Unavoidable, however, is the difficulty for novice SM researchers, who, as our findings suggest, cannot bring contributory expertise to RECs, but rather gain interactional expertise from them. This is supported by other researchers who claim “those newer to the field were struggling with knowing where to turn for guidance when making ethical research decisions” (Woodfield et al., 2013). This issue is compounded by researcher supervisors who are equally ill-equipped to offer advice about this type of research, placing greater importance on the role of the REC. RECs, rather than being reactive, must have strategies in place before these situations arise to be considered “experts” and should consult with those who possess this expertise and seek relevant training. As noted earlier in this article, the issues identified in this research are not unique to SM, but are rather a product of the fast-paced nature of scientific research more generally. RECs are often faced which novel technologies and research approaches which may present new ethical challenges. These strategies, and recommendations made below, could then potentially also be applied, if required, to the other areas of scientific research subjected to ethics review which are similarly fast-paced.

### Best Practices

Several points of action could be adopted to better equip RECs with the interactional expertise necessary to fulfill their role effectively for SM research. First, although we understand it is not always feasible to expect RECs to possess experience and expertise in all kind of research under review in these fast changing research contexts (Buchanan & Hvizdak, 2009; Henderson et al., 2013), given the complex nature of SM research ethical decision making, and the increasing use of SM data by researchers generally, RECs should ensure that at least one REC member on a committee has experience in the field of SM research. As noted above, it is also important that RECs engage in a dialogue with SM researchers and that researchers think carefully about the ethical implications of their research before review, providing sufficient context to the committee. We also do not rule out the establishment of a super-ethics committee for SM research and/or other innovative digital research platforms (Savulescu, 2017), although the logistics of such a committee would require further reflection. Although our findings suggest that there are limitations with current institutional and professional guidelines based on traditional ethical frameworks (Emanuel et al., 2008), we do not suggest that new separate guidelines are needed for SM research, as it was not the aim of this research to identify whether more guidelines are required. We have previously discussed the relevance and limitations of current guidelines for SM research ethics and refer the reader to these discussions (Samuel et al., 2019). Rather, for now, we suggest a more nuanced, contextualized application of existing guidelines, assessing applications on a case-by-case basis, in addition to the regular updating of these guidelines to reflecting the changing nature of research as we learn more about its ethical implications, would address these limitations. Participants suggested that this process was already in motion in certain institutions, and professional bodies such as British Educational Research Association (2018) have also updated their exiting guidance to include SM research. Furthermore, a number of participants questioned both the feasibility and desirability of strict formal guidance, considering the dynamic nature of the field and variety of platforms available.

### Limitations

This study has limitations. First, the exploratory nature of the research and the nature and size of the sample means that the claims that can be made are limited. For instance, despite the fact that participants came from a range of disciplinary backgrounds, it makes it difficult to make claims regarding differences between disciplines in RECs confidence and perceived ability to review SM proposals. This would be an important factor to explore in future research and would require a broader range of disciplines (e.g., representation from the Arts and Humanities and the core Sciences), and greater representations from each group.

### Research Agenda

Other factors not addressed here, but which would be interesting future directions for research, include the perspectives of SM users with regard to the use of SM data for research purposes, and differences in the impact of the specific SM platforms used in research on RECs confidence and ability to review such research. It is also important that future research explores the effectiveness of any training initiatives, especially since one of our interviewees still felt inexperienced when reviewing SM proposals (an “observer”) while being a member of the Association of Internet Researchers and having some awareness of the ethical issues.

### Educational Implications

In cases where it is not possible to implement the recommendations made above, it may be effective to engage REC members and researchers in training in the ethics of SM research, as has been proposed by others (Buchanan & Hvizdak, 2009; Woodfield et al., 2013), preferably led by researchers using SM who can share their contributory expertise. Several researchers and REC members suggested that this was already happening in their respective institutions and it is a strategy that has been used in other research contexts where ethical expertise may be lacking (Aalborg et al., 2016; Aboud et al., 2018; Ajuwon & Kass, 2008; Chen, 2003; Dubois, Dueker, Anderson, & Campbell, 2008). Such training might also take the form of online, peer reviewed, open access training modules on the ethics of SM research to complement those already hosted on platforms such as the training and resources in research ethics evaluation (TRREE; https://elearning.trree.org/) website.

Overall, REC interviewees lacked experience and expertise when reviewing SM research, and while strategies were sometimes in place to negotiate this inexperience, and increase their interactional expertise through speaking to SM research experts, researchers themselves still perceived REC lack of experience/expertise as problematic. We have suggested several steps to ensure REC interactional expertise in SM research keeps up with the pace of this fast-developing field.

## Notes

1. There are, however, an increasing number of exceptions, with a range of case studies being published which discuss the ethical issues arising from specific SM research projects (Grinyer, 2007; Lomborg, 2013; Sharkey et al., 2011; Whiteman, 2012).

2. The authors are aware that some of the evidence on which these criticisms are based is anecdotal, however, such evidence still indicates that there is an issue with regard to perceptions of REC expertise. Further support for this is provided by empirical evidence, such as that cited in this section, which demonstrates the challenges faced by RECs when reviewing research in which lack experience or expertise. We also highlight in this section how RECs deal with this perceived lack of expertise and the strategies they put in place.

3. RECs in U.K. HEIs are not directly governed by U.K. laws, although they must comply with a number of laws relevant to research ethics, such as those relating to data protection and human rights. The organization and composition of RECs are largely at the discretion of the host institution/ department, although in certain disciplines there may be funder/ professional body requirements that need to be met.

4. See Note 1.

5. The authors are aware that it is common for researchers to be critical of RECs, as discussed in Van den Hoonaard and Hamilton (2016), and that researchers may hold some bias. However, it is of value to consider their views as RECs and researchers negotiate this emerging field of SM research.
